# Examination of potential novel biochemical factors in relation to prostate cancer incidence and mortality in UK Biobank

**DOI:** 10.1038/s41416-020-01081-3

**Published:** 2020-09-23

**Authors:** Aurora Perez-Cornago, Georgina K. Fensom, Colm Andrews, Eleanor L. Watts, Naomi E. Allen, Richard M. Martin, Mieke Van Hemelrijck, Timothy J. Key, Ruth C. Travis

**Affiliations:** 1 Cancer Epidemiology Unit, Nuffield Department of Population Health, University of Oxford, Oxford, UK; 2 Clinical Trial Service Unit and Epidemiological Studies Unit, Nuffield Department of Population Health, University of Oxford, Oxford, UK; 3 MRC Integrative Epidemiology Unit (IEU), Population Health Sciences, Bristol Medical School, University of Bristol, Bristol, UK; 4 Bristol Medical School, Department of Population Health Sciences, University of Bristol, Bristol, UK; 5 National Institute for Health Research (NIHR) Bristol Biomedical Research Centre, University Hospitals Bristol NHS Foundation Trust and the University of Bristol, Bristol, UK; 6 Translational Oncology & Urology Research, School of Cancer and Pharmaceutical Sciences, King’s College London, London, UK; 7 Unit of Epidemiology, Institute of Environmental Medicine, Karolinska Institutet, Stockholm, Sweden

**Keywords:** Predictive markers, Prostate cancer, Risk factors

## Abstract

**Background:**

Although prostate cancer is a leading cause of cancer death, its aetiology is not well understood. We aimed to identify novel biochemical factors for prostate cancer incidence and mortality in UK Biobank.

**Methods:**

A range of cardiovascular, bone, joint, diabetes, renal and liver-related biomarkers were measured in baseline blood samples collected from up to 211,754 men at recruitment and in a subsample 5 years later. Participants were followed-up via linkage to health administrative datasets to identify prostate cancer cases. Hazard ratios (HRs) and 95% confidence intervals were calculated using multivariable-adjusted Cox regression corrected for regression dilution bias. Multiple testing was accounted for by using a false discovery rate controlling procedure.

**Results:**

After an average follow-up of 6.9 years, 5763 prostate cancer cases and 331 prostate cancer deaths were ascertained. Prostate cancer incidence was positively associated with circulating vitamin D, urea and phosphate concentrations and inversely associated with glucose, total protein and aspartate aminotransferase. Phosphate and cystatin-C were the only biomarkers positively and inversely, respectively, associated with risk in analyses excluding the first 4 years of follow-up. There was little evidence of associations with prostate cancer death.

**Conclusion:**

We found novel associations of several biomarkers with prostate cancer incidence. Future research will examine associations by tumour characteristics.

## Background

Prostate cancer is the second most commonly diagnosed cancer in men worldwide after lung cancer and is a leading cause of cancer death.^1^ However, its well-established risk factors age, ethnicity family history and other genetic factors are not modifiable.^2,3^ There is evidence that higher circulating insulin-like growth factor-I (IGF-I) concentrations are related to higher overall prostate cancer risk,^4,5^ and obesity has been associated with a higher risk of aggressive disease.^6^ Moreover, men with low free testosterone concentrations may have a lower risk of overall prostate cancer.^7^ However, the aetiology of prostate cancer is not well understood, and there is a need to identify novel risk factors for the disease.

There is some evidence that some non-communicable diseases are associated with prostate cancer risk. Men with diabetes or kidney disease have a lower risk of being diagnosed with prostate cancer,^8,9^ but it is unknown whether concentrations of common biochemical factors related to other non-communicable diseases, such as cardiovascular or liver disease, are also associated with prostate cancer. Due to the high cost of measuring multiple biomarkers in samples from an entire cohort, most previous studies of potential biomarkers for prostate cancer risk have been nested case-control studies of a small number of selected putative cancer biomarkers.

The UK Biobank prospective cohort study is an important resource for the study of cancer aetiology. UK Biobank has measured concentrations of multiple biomarkers known to be related to several non-communicable diseases (i.e. cardiovascular, bone and joint, diabetes, renal and liver diseases) in blood and urine samples from every participant in the cohort. This provides a unique opportunity to investigate a wide range of biomarkers, including biomarkers that have not previously been studied in relation to prostate cancer risk. We report here the first results from this cohort on the association of concentrations of blood and urine biomarkers known to be related to other non-communicable diseases with prostate cancer incidence and mortality in a large British cohort.

## Methods

### Study design and population

UK Biobank is a prospective study of >500,000 people (aged 40–69 years, including 229,000 men) designed to be a resource for research into the causes of disease in middle and old age. The study protocol and information about data access are available online (http://www.ukbiobank.ac.uk/wp-content/uploads/2011/11/UK-Biobank-Protocol.pdf) and elsewhere.^10^ All the covariates were measured at the same time as the biological samples were collected.

Briefly, persons who lived within reasonable traveling distance (∼25 km) of 1 of the 22 assessment canters across England, Wales and Scotland were identified from National Health Service (NHS) registers and invited to participate in the study between 2006–2010, resulting in a participation rate of 5.5%.^11^ At recruitment, participants provided detailed information on a range of sociodemographic, physical, lifestyle and health-related factors via self-completed touch-screen questionnaires and a computer assisted personal interview.^11^ Anthropometric measurements (standing height, weight, waist and hip circumferences) were taken by trained research clinic staff at the assessment centre, while body mass index [BMI] and percentage body fat were assessed through bioimpedance measures.^12^ A total of 211,754 men were included in these analyses; a study flowchart showing all the exclusions can be found in Supplemental Fig. 1.

### Blood and urine collection and laboratory analysis

Blood and a mid-flow urine sample (both non-fasting) were collected at recruitment from 99.7% and 96% of participants, respectively. Repeat blood and urine samples were collected in ~9000 men between August 2012 and June 2013 at the UK Biobank Co-ordinating Centre in Stockport; participants who lived within a 35 km radius were invited to attend, with an overall response rate of 21%.^13,14^ Blood and urine samples were shipped to the central processing laboratory at 4 °C prior to serum preparation, aliquoting and cryopreservation in a central working archive.^15,16^

For the current analysis, we have included 28 biochemistry markers (cardiovascular, bone, joint, diabetes, renal and liver-related biomarkers, please see Table 1 for the full biomarker list) that were measured in 229,000 men. UK Biobank did not release data on biomarkers outside of the limit of detection for each individual biomarker; therefore, extreme outliers are already not included in the dataset. Details about assay methods and quality control procedures are available online (https://biobank.ndph.ox.ac.uk/showcase/showcase/docs/serum_biochemistry.pdf and http://biobank.ctsu.ox.ac.uk/crystal/crystal/docs/urine_assay.pdf).

**Table 1:** Baseline characteristics and biochemical data of all men and men who developed prostate cancer in UK Biobank.

	All men (*N* = 211,754)	Men who developed prostate cancer (*N* = 5763)
Sociodemographic		
Age at recruitment (years), mean (SD)	56.53 (8.20)	62.16 (5.26)
<45	10.76 (23,377)	0.42 (25)
45–49	13.10 (28,456)	2.25 (133)
50–54	14.72 (31,961)	6.86 (406)
55–59	17.63 (38,298)	15.17 (898)
60–64	23.94 (51,998)	37.66 (2229)
≥65	19.85 (43,109)	37.63 (2227)
Most deprived quintile, % (*n*)	19.76 (41,787)	15.60 (898)
Black ethnicity, % (*n*)	1.49 (3139)	2.09 (120)
Not in paid/self-employment, % (*n*)	38.58 (81,705)	57.02 (3286)
Living with partner, % (*n*)	92.93 (161,896)	95.39 (4573)
Anthropometric, mean (SD)		
Height (cm)	175.64 (6.84)	175.10 (6.70)
Body fat (%)	25.24 (5.79)	25.42 (5.47)
BMI (kg/m^2^)	27.84 (4.24)	27.53 (3.79)
Lifestyle, % (*n*)		
Current cigarette smokers	12.46 (26,225)	9.23 (529)
Drinking alcohol ≥20 g per day	43.54 (91,706)	42.55 (2443)
Low physical activity (0–10 METs per week)	28.40 (58,145)	27.23 (1520)
Health history, % (n)		
Hypertension	52.22 (110,509)	59.05 (3401)
Diabetes	6.88 (14,489)	5.88 (338)
Biomarkers, mean (SD)		
Cardiovascular-related		
Cholesterol, mmol/L	5.48 (1.13)	5.42 (1.11)
LDL-cholesterol, mmol/L	3.48 (0.86)	3.43 (0.85)
HDL-cholesterol, mmol/L	1.28 (0.31)	1.30 (0.32)
Triglycerides, mmol/L	1.98 (1.15)	1.87 (1.00)
Apolipoprotein A1, g/L	1.43 (0.23)	1.45 (0.23)
Apolipoprotein B, g/L	1.03 (0.24)	1.01 (0.23)
C-reactive protein, mg/L	2.43 (4.25)	2.37 (3.91)
Lipoprotein (a), nmol/L	43.82 (48.98)	45.41 (50.02)
Bone and joint-related		
Vitamin D, nmol/L	48.49 (21.21)	51.74 (21.04)
Alkaline phosphatase, U/L	81.94 (24.16)	81.97 (29.37)
Calcium, mmol/L	2.37 (0.09)	2.37 (0.09)
Diabetes-related		
HbA1c, mmol/mol	36.49 (7.60)	36.58 (6.64)
Glucose, mmol/L	5.19 (1.41)	5.15 (1.22)
Renal-related		
Cystatin-C, mg/L	0.94 (0.18)	0.96 (0.17)
Creatinine, umol/L	81.58 (18.69)	82.83 (19.90)
Total protein, g/L	72.64 (4.08)	72.05 (4.05)
Urea, mmol/L	5.60 (1.44)	5.83 (1.50)
Phosphate, mmol/L	1.12 (0.16)	1.12 (0.16)
Urate, umol/L	354.41 (71.59)	352.48 (69.76)
Creatinine (Urine), micromole/L	10.90 (6.10)	10.58 (5.79)
Microalbumin (Urine), mg/L	11.02 (24.76)	11.25 (24.48)
* Potassium/creatinine ratio*	6.98 (3.87)	7.22 (2.84)
* Sodium/creatinine ratio*	9.67 (5.64)	9.41 (5.19)
Liver-related		
Albumin, g/L	45.54 (2.61)	45.15 (2.54)
Direct bilirubin, umol/L	2.01 (0.94)	2.04 (0.88)
Total bilirubin, umol/L	10.31 (4.92)	10.44 (5.00)
Gamma glutamyltransferase, U/L	45.66 (48.30)	43.64 (39.22)
ALT, U/L	27.52 (15.28)	25.47 (13.78)
AST, U/L	28.25 (11.39)	27.48 (12.45)

*ALT* alanine aminotransferase, *ApoA1* apolipoprotein A1, *ApoB* apolipoprotein B, *AST* aspartate aminotransferase, *BMI* body mass index, *HbA1c* haemoglobin A1c, *HDL* high-density lipoprotein, *LDL-cholesterol* low-density lipoperotein cholesterol, *METs* metabolic equivalents, *SD* standard deviation.

### Ascertainment of prostate cancer incidence and mortality

For prostate cancer incidence, the endpoint was first diagnosis of prostate cancer (International Classification of Diseases Tenth revision codes: C61^17^) or death from prostate cancer (prostate cancer mentioned on the death certificate), whichever was first. Men were followed-up for cancer incidence until the censoring dates (31 March 2016 in England and Wales and 31 October 2015 in Scotland).

For analyses of prostate cancer mortality, the endpoint was prostate cancer as the underlying cause of death recorded on the death certificate and men were followed-up until 31 January 2018 for England and Wales and 30 November 2016 for Scotland.

Cancer incidence and mortality data were provided by the NHS Digital for England and Wales and by the NHS Central Register and Information and Statistics Division for Scotland. Person-years were calculated from the date of recruitment to the date of cancer registration (first malignant neoplasm, except non-melanoma skin cancer (ICD-10 C44)), death date lost to follow-up or the censoring date, whichever occurred first.

### Statistical analysis

Logarithmically transformed concentrations of the biochemistry markers were used for all analyses to approximate a normal distribution. Cox proportional hazards models were used to calculate hazard ratios (HRs) and 95% confidence intervals (CIs) for prostate cancer incidence and death, using age as the underlying time variable. Men were categorised into fifths (for incident analyses) and thirds (for death analyses, due to the more limited number of events) of biomarker concentrations based on the distribution in the cohort. We also modelled HRs per SD higher concentrations. The minimally adjusted models were stratified by geographical region of recruitment (ten UK regions) and age (<45, 45–49, 50–54, 55–59, 60–64 and ≥65 years) at recruitment. The fully adjusted model was further adjusted for Townsend deprivation score (fifths, unknown [0.1%]), ethnic group (white, mixed background, Asian, black, other and unknown [0.7%]), height (<170, 170–174.9, 175–179.9 and ≥180 cm and unknown [0.6%]), lives with a wife or partner (no, yes), body mass index (BMI) (<25, 25–<29.9, 30–34.9 and ≥35 kg/m^2^ and unknown [0.6%]), cigarette smoking (never, former, current 1–<15 cigarettes per day, current ≥15 cigarettes per day, current but number of cigarettes per day unknown and smoking status unknown [0.7%]), physical activity (low [0–9.9 METs/week], moderate [10–49.9 METs/week] and high [≥50 METs/week], unknown [3.7%]), alcohol consumption (non-drinkers, <1–9.9, 10–19.9 and ≥20 g ethanol/day, unknown [0.6%]) and diabetes (no, yes, and unknown [0.6%]). For the vitamin D analyses, we further adjusted the fully adjusted model by month of recruitment to allow for the influence of month of blood draw on circulating concentrations. Categories of the adjustment covariates were defined a priori based on previous analyses by our group using UK Biobank data.^8^ To check for violation of the proportional hazards assumption we examined time-varying covariates and Schoenfeld residuals. This check did not indicate any such violation.

Measurement error and within-person variability when using single biomarker measurements lead to under-estimation (regression dilution bias) of potential associations between biomarker concentrations and prostate cancer risk.^18^ Repeated biomarker measures were available from a subsample of ~9000 men who had provided a second blood sample ~4–5 years after recruitment, and these were used to correct risk analyses for regression dilution bias using the McMahon–Peto method.^19^ In MacMahon’s method, individuals are grouped into fifths according to their first biomarker measurement, and the mean of the biomarker is calculated for each group at each repeat. The MacMahon’s regression dilution ration (RDR) is the ratio of the range of means at the repeat to the range of means at the first measurement.

Sensitivity analyses to reduce reverse causality were performed by repeating the analyses after excluding the first 4 years of follow-up; due to the relatively small number of deaths and limited statistical power, this sensitivity analysis was not performed for prostate cancer death. We also restricted analyses to those biomarkers measured using aliquot 1, which was the vast majority (~88% of the samples), in order to assess whether the inadvertent dilution of some aliquots had an impact on the associations.^20^ Finally, we restricted analyses to men aged ≥50 years to exclude those with very prostate cancer risk.

All analyses were performed using Stata version 14.1 (Stata Corporation, College Station, TX, USA), all tests of significance were two-sided, and to account for multiple testing the false discovery rate was controlled to 0.05 using the Benjamini–Hochberg method.^21^

## Results

### Participants’ characteristics

During the follow-up period (mean 6.9 years, SD: 1.4 for incidence; and 8.7 years, SD: 1.2 for mortality), 5968 men were diagnosed with prostate cancer and there were 331 prostate cancer deaths. Table 1 shows the characteristics of the study participants at baseline, including means and SDs for baseline biomarker measurements. Among all participants, the mean BMI was 27.8 kg/m^2^, 13% reported that they were current cigarette smokers, 43.5% reported drinking at least 20 g of alcohol per day and 28.7% of men reported being physically inactive. The mean age at diagnosis was 66 years (SD, 5.4 years). Regression dilution ratios, which measure within-person variability, are reported in Supplementary Table 1, and ranged from 0.21 to 1.03, with an average of 0.62.

**Fig. 1:**
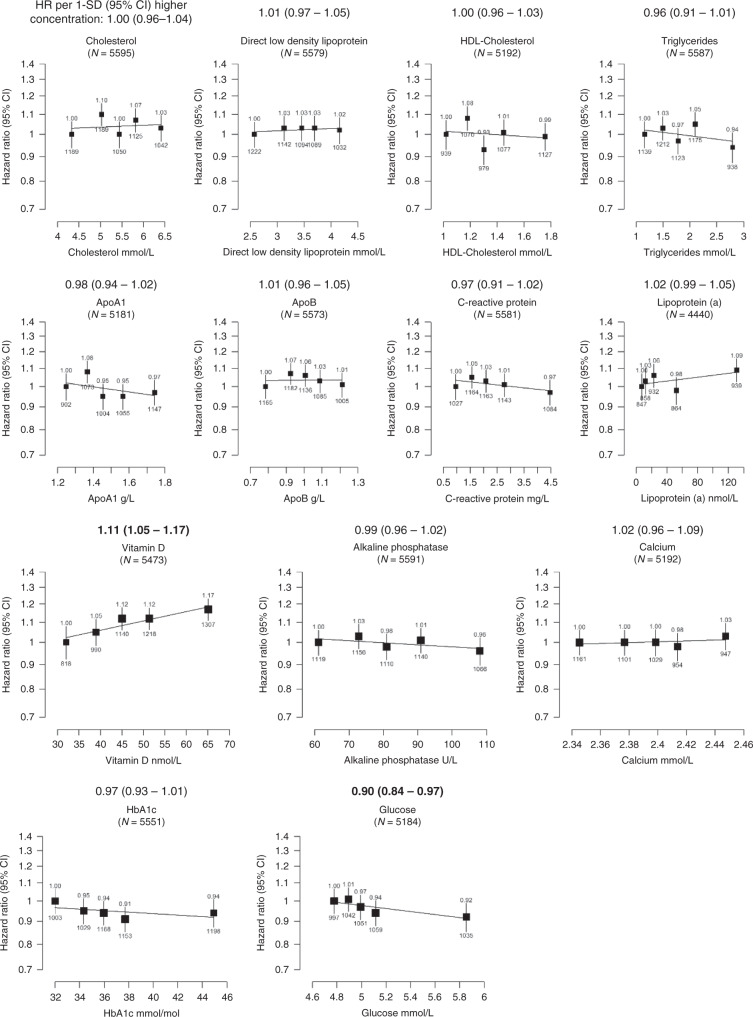
Hazard ratio of incident prostate cancer by fifths of usual serum cardiovascular-, bone-, joint- and diabetes-related biomarker concentrations in up to 205,529 men in UK Biobank. The hazard ratios above each plot are for 1-SD higher concentration of the biomarker after correction for regression dilution bias. HRs are from Cox regression analyses stratified by region and age at recruitment and adjusted for age (underlying time variable), Townsend deprivation score, ethnicity, lives with a wife or partner, BMI, smoking, physical activity, alcohol consumption and diabetes. For vitamin D analyses, the model was further adjusted for month of recruitment. Full details for each covariate are provided in the statistical section. The boxes represent the hazard ratios; the vertical lines represent the 95% CIs. The *x*-axis shows the mean concentrations of the repeat biomarker measurement within each fifth. The numbers above the vertical lines are point estimates for hazard ratios, and the numbers below are the number of prostate cancer diagnoses within each fifth. Multivariable-adjusted *P* values marked in boldface were statistically significant after allowing for multiple testing. ApoA1 apolipoprotein A1, ApoB apolipoprotein B, BMI body mass index, CI confidence intervals, HbA1c haemoglobin A1c, HDL high-density lipoprotein, HR hazard ratio, N number of prostate cancer cases.

**Fig. 2:**
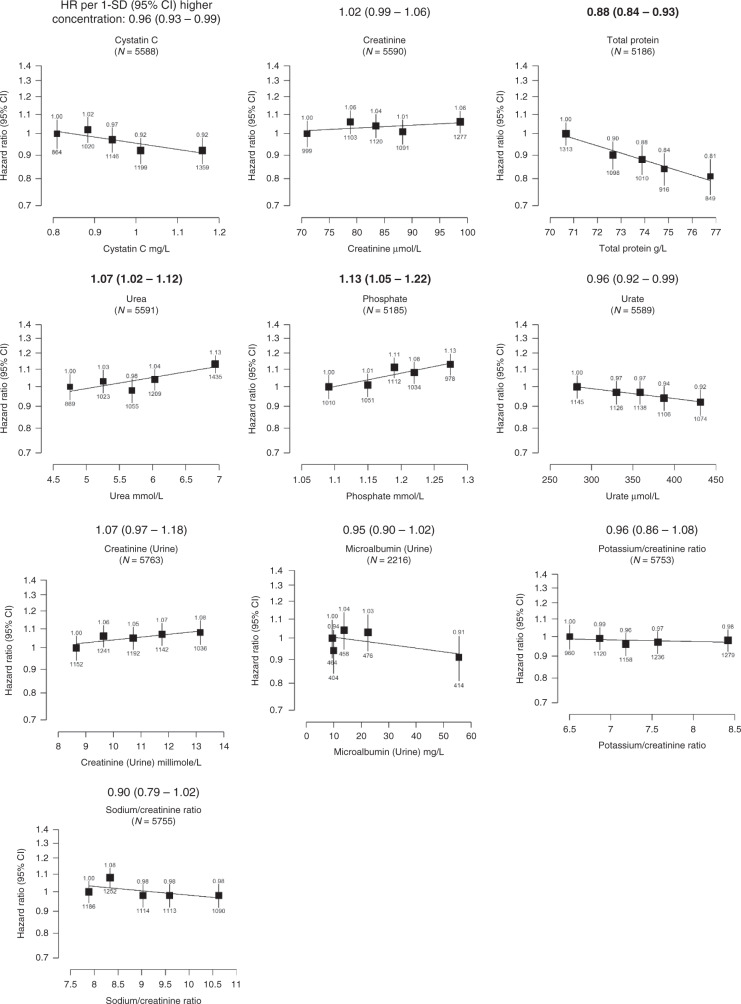
Hazard ratio of incident prostate cancer by fifths of usual serum renal-related biomarker concentrations in up to 211,754 men in UK Biobank. The hazard ratios above each plot are for 1-SD higher concentration of the biomarker after correction for regression dilution bias. HRs are from Cox regression analyses stratified by region and age at recruitment and adjusted for age (underlying time variable), Townsend deprivation score, ethnicity, lives with a wife or partner, BMI, smoking, physical activity, alcohol consumption and diabetes. Full details for each covariate are provided in the statistical section. The boxes represent the hazard ratios; the vertical lines represent the 95% CIs. The *x*-axis shows the mean concentrations of the repeat biomarker measurement within each fifth. The numbers above the vertical lines are point estimates for hazard ratios, and the numbers below are the number of prostate cancer diagnoses within each fifth. Values marked in boldface were statistically significant after allowing for multiple testing. Multivariable-adjusted *P* values marked in boldface were statistically significant after allowing for multiple testing. BMI body mass index, CI confidence intervals, HR hazard ratio, N number of prostate cancer cases.

**Fig. 3:**
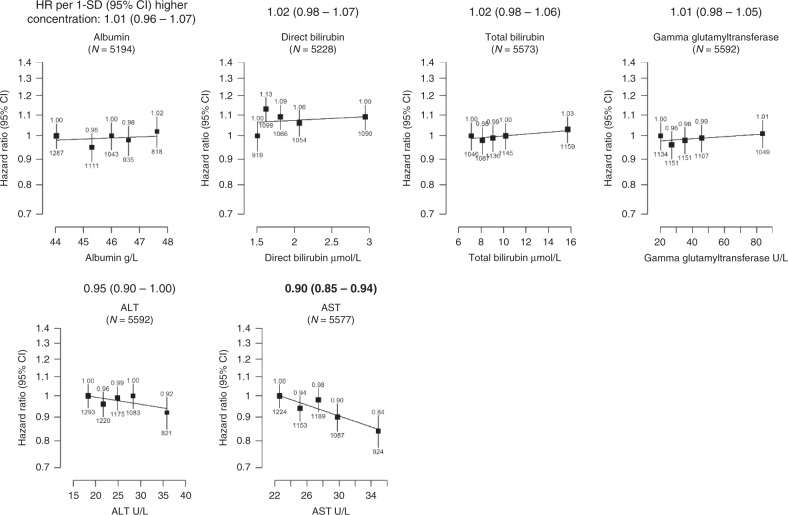
Hazard ratio of incident prostate cancer by fifths of usual serum liver-related biomarker concentrations in up to 204,621 men in UK Biobank. The hazard ratios above each plot are for 1-SD higher concentration of the biomarker after correction for regression dilution bias. HRs are from Cox regression analyses stratified by region and age at recruitment and adjusted for age (underlying time variable), Townsend deprivation score, ethnicity, lives with a wife or partner, BMI, smoking, physical activity, alcohol consumption and diabetes. Full details for each covariate are provided in the statistical section. The boxes represent the hazard ratios; the vertical lines represent the 95% CIs. The *x*-axis shows the mean concentrations of the repeat biomarker measurement within each fifth. The numbers above the vertical lines are point estimates for hazard ratios, and the numbers below are the number of prostate cancer diagnoses within each fifth. Values marked in boldface were statistically significant after allowing for multiple testing. Multivariable-adjusted *P* values marked in boldface were statistically significant after allowing for multiple testing. ALT alanine aminotransferase, AST aspartate aminotransferase, BMI body mass index, CI confidence intervals, HR hazard ratio, N number of prostate cancer cases.

The associations of concentrations of blood and urine biomarkers with prostate cancer incidence, corrected for regression dilution bias, are reported in Figs. 1–3, and associations without correction for regression dilution bias are shown in Supplementary Tables 2, 3, 4 and 5, while both uncorrected and corrected HRs for 1-SD higher circulating concentrations of each biomarker are shown in Supplementary Table 1. The associations were generally slightly greater in magnitude after adjustment for regression dilution bias (see Supplementary Table 1); the HRs and (95% CIs) below reported in the text below are the corrected results.

### Cardiovascular-related biomarkers

Figure 1 (and Supplementary table 2) shows that cardiovascular-related biomarkers [total cholesterol, low-density lipoprotein–cholesterol (LDL-c), high-density lipoprotein–cholesterol (HDL-c), triglycerides (TG), apolipoprotein A-I (ApoA-I), apolipoprotein B (ApoB), C-reactive protein (CRP) and lipoprotein a] were not related to prostate cancer diagnosis or mortality.

### Bone- and joint-related biomarkers

Higher circulating vitamin D concentrations were associated with an elevated risk of prostate cancer (HR per 1 SD increase 1.11, 95% CI 1.05–1.17, *P* trend = 0.0004) (Fig. 1 and Supplementary table 3). There was little evidence that concentrations of the other bone- and joint-related biomarkers were associated with prostate cancer incidence or death (Fig. 1 and Table 2).

### Diabetes-related biomarkers

Men with higher circulating glycated haemoglobin (HbA1c) concentration had a higher risk of dying from prostate cancer (HR per 1 SD increase 1.19, 1.02–1.39, *P* trend = 0.024; Table 2), but this association did not survive correction for multiple testing. Higher glucose concentration was associated with a lower prostate cancer risk (0.90, 0.84–0.97, *P* trend = 0.0082; Fig. 1), but was not associated with prostate cancer mortality.

**Table 2:** Hazard ratios (95% CI) for prostate cancer death by thirds (uncorrected for RDB) and one standard deviation increase (corrected for RDB) in each biomarker in UK Biobank.

Biomarker	Cases/non-cases	Thirds			*P* trend	HR per 1-SD (95% CI) higher concentrations
		1. HR (95% CI)	2. HR (95% CI)	3. HR (95% CI)		Corrected for RDB
Cardiovascular-related						
Cholesterol	318/205,211	1 (Ref)	0.85 (0.65–1.13)	1.01 (0.77–1.33)	0.70	1.03 (0.87–1.23)
LDL-cholesterol	318/204,764	1 (Ref)	1.04 (0.80–1.36)	1.01 (0.76–1.33)	0.76	1.03 (0.87–1.22)
HDL-cholesterol	297/189,439	1 (Ref)	0.88 (0.65–1.17)	0.99 (0.73–1.33)	0.35	1.07 (0.93–1.23)
Triglycerides	318/204,986	1 (Ref)	0.94 (0.71–1.23)	1.05 (0.79–1.40)	0.87	0.98 (0.79–1.22)
ApoA1	296/189,166	1 (Ref)	0.82 (0.62–1.10)	0.90 (0.67–1.21)	0.43	1.07 (0.91–1.24)
ApoB	317/203,787	1 (Ref)	1.03 (0.79–1.35)	1.07 (0.81–1.42)	0.76	1.03 (0.86–1.23)
C-reactive protein	318/204,670	1 (Ref)	0.93 (0.69–1.24)	1.08 (0.81–1.43)	0.35	1.12 (0.89–1.40)
Lipoprotein (a)	240/163,568	1 (Ref)	1.14 (0.83–1.56)	1.12 (0.82–1.54)	0.68	1.03 (0.91–1.16)
Bone- and joint-related						
Vitamin D	311/198,742	1 (Ref)	0.92 (0.69–1.22)	0.73 (0.53–1.00)	0.09	0.82 (0.65–1.03)
Alkaline phosphatase	317/205,206	1 (Ref)	1.06 (0.81–1.39)	0.96 (0.73–1.27)	0.12	1.11 (0.97–1.27)
Calcium	297/189,494	1 (Ref)	1.07 (0.81–1.41)	1.06 (0.80–1.41)	0.97	1.00 (0.76–1.31)
Diabetes-related						
HbA1c	321/203,886	1 (Ref)	1.23 (0.91–1.67)	1.33 (0.99–1.80)	0.024	1.19 (1.02–1.39)
Glucose	296/189,316	1 (Ref)	1.00 (0.74–1.35)	1.26 (0.94–1.69)	0.085	1.28 (0.97–1.69)
Renal-related						
Cystatin-C	318/205,189	1 (Ref)	1.30 (0.95–1.78)	1.20 (0.88–1.65)	0.79	1.02 (0.90–1.15)
Creatinine	318/205,099	1 (Ref)	0.87 (0.66–1.14)	0.86 (0.66–1.13)	0.35	0.93 (0.81–1.08)
Total protein	296/189,318	1 (Ref)	0.87 (0.66–1.17)	1.22 (0.93–1.61)	0.22	1.14 (0.92–1.41)
Urea	318/205,064	1 (Ref)	0.90 (0.67–1.20)	0.94 (0.71–1.23)	0.52	0.94 (0.78–1.13)
Phosphate	296/189,187	1 (Ref)	1.27 (0.96–1.67)	1.18 (0.88–1.58)	0.30	1.17 (0.87–1.58)
Urate	317/204,980	1 (Ref)	1.18 (0.90–1.55)	1.03 (0.77–1.37)	0.50	0.95 (0.82–1.10)
Microalbumin (Urine)	158/74,466	1 (Ref)	1.29 (0.88–1.89)	0.98 (0.65–1.48)	0.79	0.97 (0.77–1.22)
* Potassium/creatinine ratio (urine)*	330/210,966	1 (Ref)	0.98 (0.75–1.29)	0.91 (0.69–1.20)	0.89	0.97 (0.61–1.55)
* Sodium/creatinine ratio (urine)*	331/211,207	1 (Ref)	1.02 (0.79–1.33)	0.90 (0.69–1.18)	0.58	0.86 (0.52–1.44)
Liver-related						
Albumin	297/189,561	1 (Ref)	1.07 (0.82–1.39)	1.05 (0.79–1.41)	0.91	1.01 (0.80–1.28)
Direct bilirubin	296/191,058	1 (Ref)	1.02 (0.78–1.33)	0.68 (0.51–0.92)	0.086	0.85 (0.71–1.02)
Total bilirubin	318/204,303	1 (Ref)	0.78 (0.60–1.02)	0.68 (0.52–0.90)	0.028	0.83 (0.71–0.98)
Gamma glutamyltransferase	318/205,082	1 (Ref)	1.14 (0.85–1.52)	1.52 (1.14–2.03)	0.077	1.14 (0.99–1.31)
ALT	318/205,038	1 (Ref)	0.87 (0.67–1.15)	1.15 (0.87–1.52)	0.75	1.04 (0.82–1.31)
AST	318/204,366	1 (Ref)	1.10 (0.84–1.43)	1.02 (0.78–1.35)	0.95	1.01 (0.82–1.23)

Cox regression analysis. HR are stratified by region and age at recruitment and adjusted for age (underlying time variable), Townsend deprivation score, ethnicity, lives with a wife or partner, BMI, smoking, physical activity, alcohol consumption and diabetes. For vitamin D analyses, the model was further adjusted for month of recruitment. Full details for each covariate are provided in the statistical section.

*P* values for trend from 1-SD higher concentrations analyses. None of the associations were statistically significant after correction for multiple testing.

*ALT* alanine aminotransferase, *ApoA1* apolipoprotein A1, *ApoB* apolipoprotein B, *AST* aspartate aminotransferase, *BMI* body mass index, *HbA1c* haemoglobin A1c, *HDL* high-density lipoprotein, *HR* hazard ratio, *LDL-cholesterol* low-density lipoperotein cholesterol, *RDB* regression dilution bias.

### Renal-related biomarkers

Men with higher circulating total protein concentrations had a lower prostate cancer risk (HR per 1 SD increase 0.88, 0.84–0.93, *P* trend = 0.0001; Fig. 2). Circulating urea and phosphate concentrations were associated with an increased prostate cancer risk (1.07, 1.02–1.12, *P* trend = 0.0036; and 1.13, 1.05–1.22, *P* trend = 0.0006, respectively). Renal-related biomarkers were not associated with prostate cancer mortality.

### Liver-related biomarkers

Higher circulating aspartate aminotransferase (AST) concentration was associated with a lower prostate cancer risk (HR per 1 SD increase 0.90, 0.85–0.94, *P* trend ≤ 0.0001; Fig. 3). We also found that men with higher circulating total bilirubin concentration had a lower risk of prostate cancer death (0.83, 0.71–0.98, *P* trend = 0.028; Table 2), although this association did not survive correction for multiple testing. The other liver-related biomarkers were not related to prostate cancer diagnosis or death.

### Sensitivity analyses

After excluding the first 4 years of follow-up the 95% confidence intervals were wider and only cystatin-C and phosphate were associated with prostate cancer risk after controlling for multiple testing (HR per 1 SD increase 0.93, 0.89–0.97; and 1.07, 1.05–1.29, respectively; Table 3).

**Table 3:** Sensitivity analyses.

Biomarkers	Time to diagnosis ≥ 4 years		
	Cases/non-cases	HR (95% CI)	*P* trend
Cardiovascular-related			
Cholesterol	2713/193,818	1.07 (1.01–1.13)	0.030
LDL-cholesterol	2702/193,398	1.08 (1.02–1.15)	0.014
HDL-Cholesterol	2523/178,909	0.99 (0.94–1.04)	0.65
Triglycerides	2709/193,610	1.02 (0.95–1.10)	0.51
ApoA1	2520/178,658	0.98 (0.93–1.03)	0.44
ApoB	2702/192,454	1.08 (1.01–1.14)	0.019
C-reactive protein	2704/193,314	0.95 (0.87–1.03)	0.21
Lipoprotein (a)	2161/154,560	1.04 (1.00–1.09)	0.046
Bone- and joint-related			
Vitamin D	2647/187,640	1.04 (0.96–1.13)	0.33
Alkaline phosphatase	2709/193,813	0.97 (0.93–1.02)	0.26
Calcium	2523/178,966	1.05 (0.96–1.15)	0.31
Diabetes-related			
HbA1c	2693/192,506	0.96 (0.91–1.02)	0.23
Glucose	2522/178,796	0.87 (0.78–0.97)	0.011
Renal-related			
Cystatin-C	2711/193,798	0.93 (0.89–0.97)	**0.001**
Creatinine	2711/193,712	1.01 (0.96–1.06)	0.70
Total protein	2519/178,800	0.94 (0.88–1.01)	0.11
Urea	2711/193,678	1.01 (0.95–1.08)	0.69
Phosphate	2523/178,664	1.17 (1.05–1.29)	**0.003**
Urate	2708/193,596	1.00 (0.94–1.05)	0.88
Creatinine (Urine)	2811/199,658	1.08 (0.94–1.24)	0.26
Microalbumin (Urine)	1040/74,624	1.00 (0.92–1.10)	0.95
* Potassium/creatinine ratio (Urine)*	2804/199,224	0.91 (0.77–1.07)	0.25
* Sodium/creatinine ratio (Urine)*	2808/199,472	0.86 (0.72–1.04)	0.12
Liver-related			
Albumin	2524/179,025	1.06 (0.98–1.15)	0.14
Direct bilirubin	2517/180,458	0.99 (0.93–1.05)	0.80
Total bilirubin	2699/192,967	0.99 (0.94–1.05)	0.74
Gamma glutamyltransferase	2711/193,703	1.03 (0.98–1.09)	0.20
ALT	2711/193,651	1.03 (0.95–1.11)	0.52
AST	2702/193,023	0.91 (0.85–0.98)	0.013

Multivariable-adjusted hazard ratios (95% CI) for prostate cancer incidence excluding the first 4 years of follow-up and after correction for regression dilution bias in UK Biobank. Cox regression analysis. HR are stratified by region and age at recruitment and adjusted for age (underlying time variable), Townsend deprivation score, ethnicity, lives with a wife or partner, BMI, smoking, physical activity, alcohol consumption and diabetes. For vitamin D analyses, the model was further adjusted for month of recruitment. Full details for each covariate are provided in the statistical section.

*P* values for trend from 1-SD higher concentrations analyses. Multivariable-adjusted *P* values marked in boldface were statistically significant after allowing for multiple testing.

*ALT* alanine aminotransferase, *AST* aspartate aminotransferase, *ApoA1* apolipoprotein A1, *ApoB* apolipoprotein B, *BMI* body mass index, *CI* confidence intervals, *HbA1c* haemoglobin A1c, *HDL* high-density lipoprotein, *HR* hazard ratio, *LDL-cholesterol* low-density lipoperotein cholesterol, *SD* standard deviation.

The associations reported above did not vary when analyses were restricted to blood samples from aliquot 1 (Supplementary Table 6) or to men aged ≥50 years (Supplementary Table 7).

## Discussion

This large British prospective study with biomarkers measured in the entire cohort found several associations with possible novel biomarkers for prostate cancer incidence and mortality. The risk of being diagnosed with prostate cancer was higher in those with higher circulating concentrations of vitamin D, urea and phosphate, while risk was lower in those with higher circulating concentrations of glucose, total protein and AST. In analyses excluding the first 4 years of follow-up, cystatin-C and phosphate were associated with prostate cancer risk. After correction for multiple testing, there was little evidence of associations with prostate cancer death.

### Cardiovascular-related biomarkers

Our results are consistent with findings from a meta-analysis of 14 prospective studies that do not support an association between total cholesterol, LDL-c, HDL-c and TG concentrations and prostate cancer risk.^22^ This is in-line with a previous Mendelian randomisation study that did not find an association of genetic variants for these biomarkers with overall prostate cancer; however, that study did find some evidence that higher LDL-c and TG levels increase aggressive prostate cancer risk, and that a variant in HMGCR gene (that mimics the LDL lowering effect of statin drugs) reduces risk, although there was some evidence of pleiotropy.^23^

There are limited published prospective data on ApoA-I and ApoB in relation to prostate cancer risk. Our findings of no association of ApoA-I concentrations with risk contrast to findings from the one previous prospective analysis that found an inverse association with overall prostate cancer,^24^ while our null findings for ApoB are consistent with previous studies.^24,25^

In agreement with previous prospective studies,^26–28^ circulating CRP concentrations were not related to prostate cancer incidence or mortality in the current study.

### Bone- and joint-related biomarkers

Higher circulating vitamin D concentrations were associated with a higher risk of prostate cancer diagnosis in the current study, which is consistent with a previous individual participant meta-analysis of prospective studies;^29^ however, this association was not significant after excluding the first 4 years of follow-up. This finding may reflect detection bias; health-conscious men may have a higher dietary intake of vitamin D and/or vitamin D supplementation and higher sun exposure, and these men may be more likely to have a prostate specific antigen (PSA) test or to seek medical attention with early symptoms. Therefore, these men may have a higher risk of being diagnosed with prostate cancer.

We did not find an association between alkaline phosphatase or calcium concentrations and prostate cancer incidence and mortality. To the best of our knowledge, there is no previous prospective evidence on the association between alkaline phosphatase and prostate cancer risk, and results from previous prospective studies on circulating calcium concentration are not conclusive.^30–32^

### Diabetes-related biomarkers

In our study, higher glucose concentrations were related to a lower prostate cancer risk, although there was a suggestion of a positive association between circulating HbA1c concentration, a marker of long-term poor glycaemic control, and risk of dying from prostate cancer. However, the association with glucose was not significant in after 4 years of follow-up and the association with HbA1c did not survive correction for multiple testing. While previous prospective evidence has shown an inverse association between diabetes and prostate cancer risk,^33^ not many studies have assessed the association of diabetes with prostate cancer death. However, there is some prospective evidence that hyperglycaemia may be associated with an increased risk of fatal prostate cancer.^34^ Men with obesity are more likely to develop type II diabetes, and our findings for diabetes-related biomarkers in relation to prostate cancer risk are concordant with the current evidence on adiposity and prostate cancer, which shows an inverse association of adiposity with prostate cancer incidence but a positive association with prostate cancer death.^6^

### Renal-related biomarkers

Although there are few prospective data on kidney disease and prostate cancer risk, a previous individual patient data meta-analysis found that men with kidney disease may have a lower prostate cancer risk.^9^ Men with kidney disease are at higher risk of having a very low circulating testosterone concentration,^35^ which in turn has been associated with a lower prostate cancer risk.^7^ Findings from the present study showed that higher circulating urea and phosphate concentrations were associated with an increased prostate cancer risk, while cystatin-C and total protein circulating concentrations (both markers of kidney function) were associated with a lower risk. However, only the associations of cystatin-C and phosphate with risk were significant in the last four years of follow-up. These results may appear contradictory as all of these are biomarkers of renal disease; however, these biomarkers reflect different kidney functions. For example, elevated cystatin-C is a marker of impaired glomerular filtration rate, while phosphate concentrations are regulated by intestinal absorption, kidney excretion and bone metabolism.^36^ To the best of our knowledge, no previous prospective study has assessed the association of circulating phosphate with prostate cancer risk; however, a previous prospective study found a positive association between phosphorous intake and risk of lethal prostate cancer risk^37^ and there seems to be a positive association between the consumption of foods with phosphate additives and higher circulating concentrations.^38^ More studies are needed to clarify the association between circulating phosphate concentrations and prostate cancer risk.

### Liver-related biomarkers

This study found that higher circulating concentrations of AST were associated with a lower prostate cancer risk, although there was no association after excluding the first 4 years of follow-up. This is, to our knowledge, the first prospective study of the association between these liver-related biomarkers and prostate cancer risk and more studies with longer follow-up are needed to further investigate this possible association. Only three previous prospective studies have examined the association between non-alcoholic fatty liver disease and prostate cancer risk, and the results were inconsistent.^39–41^

### Study strengths and limitations

To the best of our knowledge, this is the largest prospective study looking at a wide range of biomarker measurements and prostate cancer incidence and mortality data. The UK Biobank has measured a panel of biomarkers in blood samples from the entire cohort, including biomarkers that have not been previously studied in prostate cancer analyses. Moreover, repeated measurements of biomarkers were available for a subsample of repeat blood specimens collected 4 years after recruitment, which has made it possible for us to adjust estimates of associations between biomarkers and risk for regression dilution bias.^18^ In addition, this is a relatively young cohort, which permits examination of factors related to earlier onset prostate cancer. Many covariates, such as adiposity measurements or blood pressure, were assessed by trained research clinic staff instead of being self-reported.

This study also has several limitations. Although UK Biobank includes participants from multiple regions across the UK, including deprived areas, it is not a representative sample of the whole UK population^11^ and this cohort may have suffered from selection bias,^42^ although the direction of risk factor associations in the UK Biobank seem to be generalisable.^43^ As in every observational study, there may be some residual confounding by unmeasured risk factors. Some of the results may be influenced by detection bias, as comorbidities may affect both biomarker concentrations and prostate specific antigen test attendance and/or results. Moreover, the blood samples of the current study are non-fasting and we do not have 24-h urine samples, and therefore, some biological measurements may have been affected by time since last meal. While we performed sensitivity analyses excluding the first 4 years of follow-up some of the associations may be due to reversed causality, as the follow-up is relatively short. Moreover, due to the small number of prostate cancer deaths we had limited power to find associations. While we have hypotheses for some of the biomarkers, we do not have a hypothesis for others as they have not previously been assessed in relation to prostate cancer, and some of the analyses using these biomarkers were exploratory. Finally, as in other epidemiological studies, there may be some misclassification of the underlying cause of death; furthermore, information on tumour characteristics, such as tumour stage and Gleason grade, is currently unavailable in the UK Biobank.

In conclusion, we have reported a range of possible novel biomarkers for prostate cancer incidence and mortality in this large UK prospective study. Since some of these analyses were exploratory, these results should be interpreted carefully. Future analyses will include research by tumour characteristics, which will help to determine whether these associations may be due to differences in the likelihood of being diagnosed and/or differences in the risk of developing clinically important prostate cancer.

## Supplementary information

Supplemental material

## Data Availability

This work has been conducted using the UK Biobank Resource under Application Number 3282. Bona-fide researchers can apply to use the UK Biobank dataset by registering and applying at http://www.ukbiobank.ac.uk/register-apply

## References

[1] Ferlay J, Soerjomataram I, Dikshit R, Eser S, Mathers C, Rebelo M (2015). Cancer incidence and mortality worldwide: sources, methods and major patterns in GLOBOCAN 2012. Int. J. Cancer.

[2] WCRF/AICR. *World Cancer Research Fund International/American Institute for Cancer Research Continuous Update Project Report: Diet, Nutrition, Physical Activity, and Prostate Cancer.* http://www.wcrf.org/sites/default/files/Prostate-Cancer-SLR-2014.pdf (2014).

[3] Cuzick J, Thorat MA, Andriole G, Brawley OW, Brown PH, Culig Z (2014). Prevention and early detection of prostate cancer. Lancet Oncol..

[4] Travis RC, Appleby PN, Martin RM, Holly JM, Albanes D, Black A (2016). A meta-analysis of individual participant data reveals an association between circulating levels of IGF-I and prostate cancer risk. Cancer Res..

[5] Watts, E. L., Goldacre, R., Key, T. J., Allen, N. E., Travis, R. C. & Perez-Cornago, A. Hormone-related diseases and prostate cancer: an English national record linkage study. *Int. J. Cancer*10.1002/ijc.32808 (2019). 10.1002/ijc.32808 PMC7318262 31755099

[6] Perez-Cornago A, Appleby PN, Pischon T, Tsilidis KK, Tjonneland A, Olsen A (2017). Tall height and obesity are associated with an increased risk of aggressive prostate cancer: results from the EPIC cohort study. BMC Med..

[7] Watts EL, Appleby PN, Perez-Cornago A, Bueno-de-Mesquita HB, Chan JM, Chen C (2018). Low free testosterone and prostate cancer risk: a collaborative analysis of 20 prospective studies. Eur. Urol..

[8] Perez-Cornago A, Key TJ, Allen NE, Fensom GK, Bradbury KE, Martin RM (2017). Prospective investigation of risk factors for prostate cancer in the UK Biobank cohort study. Br. J. Cancer.

[9] Wong G, Staplin N, Emberson J, Baigent C, Turner R, Chalmers J (2016). Chronic kidney disease and the risk of cancer: an individual patient data meta-analysis of 32,057 participants from six prospective studies. BMC Cancer.

[10] Sudlow C, Gallacher J, Allen N, Beral V, Burton P, Danesh J (2015). UK biobank: an open access resource for identifying the causes of a wide range of complex diseases of middle and old age. PLoS Med..

[11] Fry A, Littlejohns TJ, Sudlow C, Doherty N, Adamska L, Sprosen T (2017). Comparison of sociodemographic and health-related characteristics of UK Biobank participants with those of the general population. Am. J. Epidemiol..

[12] UK Biobank. *UK Biobank Anthropometry*. http://biobank.ctsu.ox.ac.uk/crystal/docs/Anthropometry.pdf (2014).

[13] UK Biobank. *Repeat assessment data* https://biobank.ctsu.ox.ac.uk/~bbdatan/Repeat_assessment_doc_v1.0.pdf (2013).

[14] Sheard, S. M. & Froggatt, J. *UK Biobank Haematology Data Companion Document [Internet]*. https://biobank.ctsu.ox.ac.uk/crystal/docs/haematology.pdf (2017).

[15] Elliott P, Peakman TC, Biobank UK (2008). The UK Biobank sample handling and storage protocol for the collection, processing and archiving of human blood and urine. Int. J. Epidemiol..

[16] UK Biobank. *Protocol for a Large-scale Prospective Epidemiological Resource* [Internet]. http://www.ukbiobank.ac.uk/wp-content/uploads/2011/11/UK-Biobank-Protocol.pdf (2007).

[17] WHO. *International statistical classification of diseases and related health problems*. 10th revision. http://apps.who.int/classifications/icd10/browse/2010/en (2010).

[18] Clarke R, Shipley M, Lewington S, Youngman L, Collins R, Marmot M (1999). Underestimation of risk associations due to regression dilution in long-term follow-up of prospective studies. Am. J. Epidemiol..

[19] MacMahon S, Peto R, Cutler J, Collins R, Sorlie P, Neaton J (1990). Blood pressure, stroke, and coronary heart disease. Part 1, Prolonged differences in blood pressure: prospective observational studies corrected for the regression dilution bias. Lancet.

[20] UK Biobank. *Biomarker assay quality procedures: approaches used to minimise systematic and random errors (and the wider epidemiological implications). Secondary Biomarker assay quality procedures: approaches used to minimise systematic and random errors (and the wider epidemiological implications)*. http://biobank.ctsu.ox.ac.uk/showcase/docs/biomarker_issues.pdf (2019).

[21] Benjamini Y, Hochberg Y (1995). Controlling the false discovery rate—a -practical and powerful approach to multiple testing. J. R. Stat. Soc. B.

[22] YuPeng L, YuXue Z, PengFei L, Cheng C, YaShuang Z, DaPeng L (2015). Cholesterol levels in blood and the risk of prostate cancer: a meta-analysis of 14 prospective studies. Cancer Epidemiol. Biomark. Prev..

[23] Bull CJ, Bonilla C, Holly JM, Perks CM, Davies N, Haycock P (2016). Blood lipids and prostate cancer: a Mendelian randomization analysis. Cancer Med..

[24] Van Hemelrijck M, Walldius G, Jungner I, Hammar N, Garmo H, Binda E (2011). Low levels of apolipoprotein A-I and HDL are associated with risk of prostate cancer in the Swedish AMORIS study. Cancer Causes Control.

[25] His M, Zelek L, Deschasaux M, Pouchieu C, Kesse-Guyot E, Hercberg S (2014). Prospective associations between serum biomarkers of lipid metabolism and overall, breast and prostate cancer risk. Eur. J. Epidemiol..

[26] Siemes C, Visser LE, Coebergh JW, Splinter TA, Witteman JC, Uitterlinden AG (2006). C-reactive protein levels, variation in the C-reactive protein gene, and cancer risk: the Rotterdam Study. J. Clin. Oncol..

[27] Stark JR, Li H, Kraft P, Kurth T, Giovannucci EL, Stampfer MJ (2009). Circulating prediagnostic interleukin-6 and C-reactive protein and prostate cancer incidence and mortality. Int. J. Cancer.

[28] Arthur R, Williams R, Garmo H, Holmberg L, Stattin P, Malmstrom H (2018). Serum inflammatory markers in relation to prostate cancer severity and death in the Swedish AMORIS study. Int. J. Cancer.

[29] Travis RC, Perez-Cornago A, Appleby PN, Albanes D, Joshu CE, Lutsey PL (2019). A collaborative analysis of individual participant data from 19 prospective studies assesses circulating vitamin D and prostate cancer risk. Cancer Res..

[30] Skinner HG, Schwartz GG (2008). Serum calcium and incident and fatal prostate cancer in the National Health and Nutrition Examination Survey. Cancer Epidemiol. Biomark. Prev..

[31] Brandstedt J, Almquist M, Manjer J, Malm J (2012). Vitamin D, PTH, and calcium and the risk of prostate cancer: a prospective nested case-control study. Cancer Causes Control.

[32] Yarmolinsky J, Berryman K, Langdon R, Bonilla C, consortium P, Davey Smith G (2018). Mendelian randomization does not support serum calcium in prostate cancer risk. Cancer Causes Control.

[33] Tsilidis KK, Kasimis JC, Lopez DS, Ntzani EE, Ioannidis JP (2015). Type 2 diabetes and cancer: umbrella review of meta-analyses of observational studies. BMJ.

[34] Marrone MT, Selvin E, Barber JR, Platz EA, Joshu CE (2019). Hyperglycemia, classified with multiple biomarkers simultaneously in men without diabetes, and risk of fatal prostate cancer. Cancer Prev. Res. (Philos.).

[35] Carrero JJ, Qureshi AR, Nakashima A, Arver S, Parini P, Lindholm B (2011). Prevalence and clinical implications of testosterone deficiency in men with end-stage renal disease. Nephrol. Dial. Transpl..

[36] Goretti Penido M, Alon US (2012). Phosphate homeostasis and its role in bone health. Pediatr. Nephrol..

[37] Wilson KM, Shui IM, Mucci LA, Giovannucci E (2015). Calcium and phosphorus intake and prostate cancer risk: a 24-y follow-up study. Am. J. Clin. Nutr..

[38] Moore LW, Nolte JV, Gaber AO, Suki WN (2015). Association of dietary phosphate and serum phosphorus concentration by levels of kidney function. Am. J. Clin. Nutr..

[39] Choi YJ, Lee DH, Han KD, Yoon H, Shin CM, Park YS (2018). Is nonalcoholic fatty liver disease associated with the development of prostate cancer? A nationwide study with 10,516,985 Korean men. PLoS ONE.

[40] Choi WM, Lee JH, Yoon JH, Kwak C, Lee YJ, Cho YY (2014). Nonalcoholic fatty liver disease is a negative risk factor for prostate cancer recurrence. Endocr. Relat. Cancer.

[41] Arase Y, Kobayashi M, Suzuki F, Suzuki Y, Kawamura Y, Akuta N (2012). Difference in malignancies of chronic liver disease due to non-alcoholic fatty liver disease or hepatitis C in Japanese elderly patients. Hepatol. Res..

[42] Munafo MR, Tilling K, Taylor AE, Evans DM, Smith GD (2018). Collider scope: when selection bias can substantially influence observed associations. Int. J. Epidemiol..

[43] Batty GD, Gale CR, Kivimaki M, Deary IJ, Bell S (2020). Comparison of risk factor associations in UK Biobank against representative, general population based studies with conventional response rates: prospective cohort study and individual participant meta-analysis. BMJ.

