# Supplementary material for: Examination of potential novel biochemical factors in relation to prostate cancer incidence and mortality in UK Biobank
Source: Br J Cancer. 2020 Sep 23;123(12):1808–17. doi: 10.1038/s41416-020-01081-3 (PMC7722733; doi:10.1038/s41416-020-01081-3)
Supplement: Supplementary file 1 — Supplemental material [file 41416_2020_1081_MOESM1_ESM.pdf]

Supplementary Table 1: Associations of serum biomarker concentrations with prostate cancer incidence and mortality in UK Biobank, with and without correction for regression dilution bias in up to 211,754 men in UK Biobank.

Biomarker	Mean concentration at baseline (SD)*	Mean concentration at resurvey (SD)*	MacMahon-Peto regression ratio†	HR per 1-SD (95% CI) higher concentrations			
				Incidence		Mortality	
				Uncorrected for RDB	Corrected for RDB	Uncorrected for RDB	Corrected for RDB
**Cardiovascular-related**							
Cholesterol, mmol/L	5.48 (1.13)	5.48 (1.13)	0.67	1.00 (0.97 - 1.03)	1.00 (0.96 - 1.04)	1.02 (0.91 - 1.15)	1.03 (0.87 - 1.23)
LDL-cholesterol, mmol/L	3.48 (0.86)	3.48 (0.86)	0.66	1.01 (0.98 - 1.04)	1.01 (0.97 - 1.05)	1.02 (0.91 - 1.14)	1.03 (0.87 - 1.22)
HDL-Cholesterol, mmol/L	1.28 (0.31)	1.28 (0.31)	0.89	1.00 (0.97 - 1.03)	1.00 (0.96 - 1.03)	1.06 (0.94 - 1.20)	1.07 (0.93 - 1.23)
Triglycerides, mmol/L	1.98 (1.15)	1.98 (1.15)	0.57	0.98 (0.95 - 1.01)	0.96 (0.91 - 1.01)	0.99 (0.88 - 1.12)	0.98 (0.79 - 1.22)
ApoA1, g/L	1.43 (0.23)	1.43 (0.23)	0.80	0.98 (0.95 - 1.01)	0.98 (0.94 - 1.02)	1.05 (0.93 - 1.19)	1.07 (0.91 - 1.24)
ApoB, g/L	1.03 (0.24)	1.03 (0.24)	0.64	1.00 (0.98 - 1.03)	1.01 (0.96 - 1.05)	1.02 (0.91 - 1.14)	1.03 (0.86 - 1.23)
C-reactive protein, mg/L	2.43 (4.25)	2.43 (4.25)	0.51	0.98 (0.96 - 1.01)	0.97 (0.91 - 1.02)	1.06 (0.94 - 1.19)	1.12 (0.89 - 1.40)
Lipoprotein (a), nmol/L	43.82 (48.98)	43.82 (48.98)	1.03	1.02 (0.99 - 1.06)	1.02 (0.99 - 1.05)	1.03 (0.90 - 1.17)	1.03 (0.91 - 1.16)
**Bone and joint-related**							
Vitamin D, nmol/L	48.49 (21.21)	48.49 (21.21)	0.58	1.06 (1.03 - 1.10)	1.11 (1.05 - 1.17)	0.89 (0.78 - 1.02)	0.82 (0.65 - 1.03)
Alkaline phosphatase, U/L	81.94 (24.16)	81.94 (24.16)	0.83	0.99 (0.96 - 1.02)	0.99 (0.96 - 1.02)	1.09 (0.98 - 1.22)	1.11 (0.97 - 1.27)
Calcium, nmol/L	2.37 (0.09)	2.37 (0.09)	0.42	1.01 (0.98 - 1.04)	1.02 (0.96 - 1.09)	1.00 (0.89 - 1.12)	1.00 (0.76 - 1.31)
Diabetes-related							
HbA1c, mmol/mol	36.49 (7.60)	36.49 (7.60)	0.85	0.97 (0.94 - 1.01)	0.97 (0.93 - 1.01)	1.16 (1.02 - 1.32)	1.19 (1.02 - 1.39)
Glucose, mmol/L	5.19 (1.41)	5.19 (1.41)	0.43	0.96 (0.93 - 0.99)	0.90 (0.84 - 0.97)	1.11 (0.99 - 1.26)	1.28 (0.97 - 1.69)
**Renal-related**							
Cystatin C, mg/L	0.94 (0.18)	0.94 (0.18)	0.94	0.97 (0.94 - 0.99)	0.96 (0.93 - 0.99)	1.02 (0.91 - 1.14)	1.02 (0.90 - 1.15)
Creatinine, umol/L	81.58 (18.69)	81.58 (18.69)	0.79	1.02 (0.99 - 1.04)	1.02 (0.99 - 1.06)	0.95 (0.85 - 1.06)	0.93 (0.81 - 1.08)
Total protein, g/L	72.64 (4.08)	72.64 (4.08)	0.55	0.93 (0.91 - 0.96)	0.88 (0.84 - 0.93)	1.07 (0.96 - 1.21)	1.14 (0.92 - 1.41)
Urea, mmol/L	5.60 (1.44)	5.60 (1.44)	0.62	1.04 (1.01 - 1.07)	1.07 (1.02 - 1.12)	0.96 (0.86 - 1.08)	0.94 (0.78 - 1.13)
Phosphate, mmol/L	1.12 (0.16)	1.12 (0.16)	0.41	1.05 (1.02 - 1.08)	1.13 (1.05 - 1.22)	1.07 (0.94 - 1.21)	1.17 (0.87 - 1.58)
Urate, umol/L	354.41 (71.59)	354.41 (71.59)	0.77	0.97 (0.94 - 1.00)	0.96 (0.92 - 0.99)	0.96 (0.86 - 1.08)	0.95 (0.82 - 1.10)
Creatinine (Urine), micromole/L	10.90 (6.10)	10.90 (6.10)	0.28	1.02 (0.99 - 1.05)	1.07 (0.97 - 1.18)	1.12 (1.00 - 1.26)	1.49 (0.99 - 2.24)
Microalbumin (Urine), mg/L	11.02 (24.76)	11.02 (24.76)	0.60	0.97 (0.93 - 1.01)	0.95 (0.90 - 1.02)	0.98 (0.83 - 1.15)	0.97 (0.77 - 1.22)
Potassium/creatinine ratio	6.98 (3.87)	6.98 (3.87)	0.25	0.99 (0.96 - 1.02)	0.96 (0.86 - 1.08)	0.99 (0.88 - 1.12)	0.97 (0.61 - 1.55)
Sodium/creatinine ratio	9.67 (5.64)	9.67 (5.64)	0.21	0.98 (0.95 - 1.00)	0.90 (0.79 - 1.02)	0.97 (0.87 - 1.08)	0.86 (0.52 - 1.44)
**Liver-related**							
Albumin, g/L	45.54 (2.61)	45.54 (2.61)	0.51	1.01 (0.98 - 1.04)	1.01 (0.96 - 1.07)	1.01 (0.89 - 1.13)	1.01 (0.80 - 1.28)
Direct bilirubin, umol/L	2.01 (0.94)	2.01 (0.94)	0.67	1.02 (0.99 - 1.04)	1.02 (0.98 - 1.07)	0.90 (0.80 - 1.01)	0.85 (0.71 - 1.02)
Total bilirubin, umol/L	10.31 (4.92)	10.31 (4.92)	0.73	1.01 (0.99 - 1.04)	1.02 (0.98 - 1.06)	0.87 (0.78 - 0.99)	0.83 (0.71 - 0.98)
Gamma glutamyltransferase, U/L	45.66 (48.30)	45.66 (48.30)	0.80	1.01 (0.98 - 1.04)	1.01 (0.98 - 1.05)	1.11 (0.99 - 1.24)	1.14 (0.99 - 1.31)
ALT, U/L	27.52 (15.28)	27.52 (15.28)	0.52	0.97 (0.94 - 1.00)	0.95 (0.90 - 1.00)	1.02 (0.90 - 1.15)	1.04 (0.82 - 1.31)
AST, U/L	28.25 (11.39)	28.25 (11.39)	0.57	0.94 (0.91 - 0.97)	0.90 (0.85 - 0.94)	1.00 (0.90 - 1.13)	1.01 (0.82 - 1.23)

Cox regression analysis. HR are stratified by region and age at recruitment and adjusted for age (underlying time variable), Townsend deprivation score, ethnicity, lives with a wife or partner, BMI, smoking, physical activity, alcohol consumption, and diabetes. For vitamin D analyses, the model was further adjusted for month of recruitment. Full details for each covariate are provided in the statistical section.

*Values are restricted to men with both baseline and repeat measurements.

†R_r/R_b where:

R_r=difference between the mean value at resurvey in the highest fifth of the distribution at baseline and mean value at resurvey in the lowest fifth of the distribution at baseline.

R_b= difference between the mean value in the highest fifth of the distribution at baseline and the mean value in the lowest fifth of the distribution at baseline.

Abbreviations: ALT, alanine aminotransferase; AST, aspartate aminotransferase; ApoA1, apolipoprotein A1; ApoB, apolipoprotein B; BMI, body mass index; CI, confidence interval; HbA1c, hemoglobin A1c; HDL, high-density lipoprotein; HR, hazard ratio; LDL-cholesterol, low-density lipoperotein cholesterol; RDB=regression dilution bias SD, standard deviation

Supplementary table 2. Hazard ratios (95% CI) for prostate cancer by fifths of cardiovascular-related biomarkers without correction for regression dilution bias in up to 205,529 men in UK Biobank.

	Fifths					P-trend
**Biomarkers**	**1**	**2**	**3**	**4**	**5**	
Cholesterol						
Cases/controls	1189/39938	1189/39904	1050/40090	1125/39974	1042/40028	
Model 1, HR (95% CI)	1 (Ref)	1.17 (1.08-1.27)	1.08 (1.00-1.18)	1.17 (1.08-1.27)	1.13 (1.04-1.23)	0.0051
Model 2, HR (95% CI)	1 (Ref)	1.10 (1.01-1.19)	1.00 (0.92-1.09)	1.07 (0.98-1.16)	1.03 (0.94-1.12)	0.9727
LDL-cholesterol						
Cases/controls	1222/39818	1142/39911	1094/39933	1089/39921	1032/39920	
Model 1, HR (95% CI)	1 (Ref)	1.09 (1.01-1.19)	1.11 (1.03-1.21)	1.13 (1.04-1.23)	1.11 (1.02-1.21)	0.0013
Model 2, HR (95% CI)	1 (Ref)	1.03 (0.95-1.12)	1.03 (0.94-1.12)	1.03 (0.95-1.13)	1.02 (0.93-1.11)	0.5796
HDL-Cholesterol						
Cases/controls	939/37083	1070/36927	979/37045	1077/36677	1127/36812	
Model 1, HR (95% CI)	1 (Ref)	1.14 (1.04-1.24)	1.02 (0.93-1.12)	1.12 (1.02-1.22)	1.11 (1.02-1.22)	0.0107
Model 2, HR (95% CI)	1 (Ref)	1.08 (0.98-1.17)	0.93 (0.85-1.02)	1.01 (0.92-1.10)	0.99 (0.90-1.09)	0.8438
Triglycerides						
Cases/controls	1139/40003	1212/39797	1123/39932	1175/39867	938/40118	
Model 1, HR (95% CI)	1 (Ref)	1.01 (0.93-1.09)	0.93 (0.86-1.01)	0.99 (0.92-1.08)	0.86 (0.79-0.94)	0.0001
Model 2, HR (95% CI)	1 (Ref)	1.03 (0.95-1.12)	0.97 (0.89-1.05)	1.05 (0.97-1.15)	0.94 (0.86-1.03)	0.1133
Apolipoprotein A						
Cases/controls	902/37071	1073/36910	1004/36964	1055/36810	1147/36526	
Model 1, HR (95% CI)	1 (Ref)	1.13 (1.03-1.23)	1.02 (0.93-1.11)	1.04 (0.95-1.14)	1.07 (0.98-1.17)	0.2433
Model 2, HR (95% CI)	1 (Ref)	1.08 (0.99-1.18)	0.95 (0.86-1.04)	0.95 (0.87-1.04)	0.97 (0.88-1.07)	0.2961
Apolipoprotein B						
Cases/controls	1165/39661	1182/39916	1136/39562	1085/39699	1005/39693	
Model 1, HR (95% CI)	1 (Ref)	1.11 (1.03-1.21)	1.12 (1.03-1.22)	1.11 (1.02-1.20)	1.08 (0.99-1.18)	0.0315
Model 2, HR (95% CI)	1 (Ref)	1.07 (0.98-1.16)	1.06 (0.97-1.15)	1.03 (0.95-1.13)	1.01 (0.93-1.10)	0.7983
C-reactive protein						
Cases/controls	1027/40456	1164/39935	1163/39649	1143/39498	1084/39869	
Model 1, HR (95% CI)	1 (Ref)	1.03 (0.95-1.12)	0.99 (0.91-1.08)	0.95 (0.87-1.04)	0.89 (0.82-0.97)	0.0006
Model 2, HR (95% CI)	1 (Ref)	1.05 (0.96-1.14)	1.03 (0.94-1.12)	1.01 (0.92-1.10)	0.97 (0.89-1.06)	0.2449
Lipoprotein (a)						
Cases/controls	847/32259	858/31566	932/31824	864/31898	939/31821	
Model 1, HR (95% CI)	1 (Ref)	1.04 (0.94-1.14)	1.08 (0.98-1.19)	1.01 (0.92-1.11)	1.13 (1.03-1.24)	0.0160
Model 2, HR (95% CI)	1 (Ref)	1.03 (0.94-1.13)	1.06 (0.97-1.17)	0.98 (0.89-1.08)	1.09 (0.99-1.20)	0.1129

Cox regression analysis.

Model 1: HR are stratified by region (ten UK cancer registry regions) and age at recruitment (< 45, 45 – 49, 50 – 54, 55 – 59, 60 – 64, ≥ 65 years) and adjusted for age (underlying time variable).

Model 2: HR are stratified by region and age at recruitment and adjusted for age (underlying time variable), Townsend deprivation score, ethnicity, lives with a wife or partner, BMI, smoking, physical activity, alcohol consumption, and diabetes. Full details for each covariate are provided in the statistical section.

P-values for trend from 1-SD higher concentrations analyses. Multivariable-adjusted P-values marked in boldface were statistically significant after allowing for multiple testing.

Abbreviations: ApoA1, apolipoprotein A1; ApoB, apolipoprotein B; CI, confidence intervals; BMI, body mass index; HDL, high-density lipoprotein; HR, hazard ratio; LDL-cholesterol, low-density lipoperotein cholesterol.

Supplementary table 3. Hazard ratios (95% CI) for prostate cancer by fifths of bone-, joint-, and diabetes-related biomarkers without correction for regression dilution bias in up to 205,529 men in UK Biobank.

	Fifths					P-trend
**Biomarkers**	**1**	**2**	**3**	**4**	**5**	
**Bone and joint-related**						
Vitamin D						
Cases/controls	818/39163	990/38883	1140/38767	1218/38451	1307/38316	
Model 1, HR (95% CI)	1 (Ref)	1.07 (0.98-1.18)	1.15 (1.05-1.26)	1.16 (1.06-1.27)	1.22 (1.11-1.33)	<0.0001
Model 2, HR (95% CI)	1 (Ref)	1.05 (0.95-1.15)	1.12 (1.01-1.23)	1.12 (1.02-1.24)	1.17 (1.06-1.29)	**0.0004**
Alkaline phosphatase						
Cases/controls	1119/40193	1156/39990	1110/39826	1140/40111	1066/39812	
Model 1, HR (95% CI)	1 (Ref)	1.03 (0.94-1.11)	0.97 (0.89-1.06)	0.99 (0.91-1.07)	0.92 (0.85-1.00)	0.0664
Model 2, HR (95% CI)	1 (Ref)	1.03 (0.95-1.12)	0.98 (0.90-1.07)	1.01 (0.93-1.10)	0.96 (0.88-1.04)	0.4358
Calcium						
Cases/controls	1161/36887	1101/37346	1029/36929	954/36738	947/36699	
Model 1, HR (95% CI)	1 (Ref)	1.00 (0.92-1.09)	1.00 (0.92-1.09)	0.98 (0.90-1.07)	1.02 (0.93-1.11)	0.7034
Model 2, HR (95% CI)	1 (Ref)	1.00 (0.92-1.08)	1.00 (0.92-1.09)	0.98 (0.90-1.07)	1.03 (0.94-1.12)	0.5262
**Diabetes-related**						
HbA1c						
Cases/controls	1003/41231	1029/38965	1168/40884	1153/37937	1198/39639	
Model 1, HR (95% CI)	1 (Ref)	0.94 (0.86-1.03)	0.92 (0.85-1.00)	0.88 (0.81-0.96)	0.80 (0.74-0.87)	<0.0001
Model 2, HR (95% CI)	1 (Ref)	0.95 (0.87-1.03)	0.94 (0.86-1.02)	0.91 (0.84-0.99)	0.94 (0.85-1.03)	0.1330
Glucose						
Cases/controls	997/36977	1042/36844	1051/36963	1059/36773	1035/36871	
Model 1, HR (95% CI)	1 (Ref)	1.01 (0.93-1.10)	0.98 (0.90-1.07)	0.94 (0.86-1.02)	0.84 (0.77-0.91)	<0.0001
Model 2, HR (95% CI)	1 (Ref)	1.01 (0.92-1.10)	0.97 (0.89-1.06)	0.94 (0.86-1.03)	0.92 (0.84-1.01)	**0.0082**

Cox regression analysis.

Model 1: HR are stratified by region (ten UK cancer registry regions) and age at recruitment (< 45, 45 – 49, 50 – 54, 55 – 59, 60 – 64, ≥ 65 years) and adjusted for age (underlying time variable).

Model 2: HR are stratified by region and age at recruitment and adjusted for age (underlying time variable), Townsend deprivation score, ethnicity, lives with a wife or partner, BMI, smoking, physical activity, alcohol consumption, and diabetes. For vitamin D analyses, the model was further adjusted for month of recruitment. Full details for each covariate are provided in the statistical section.

P-values for trend from 1-SD higher concentrations analyses. Multivariable-adjusted P-values marked in boldface were statistically significant after allowing for multiple testing.

Abbreviations: HbA1c, hemoglobin A1c; CI, confidence intervals; BMI, body mass index; HR, hazard ratio.

Supplementary table 4. Multivariable-adjusted hazard ratios (95% CI) for prostate cancer by fifths of renal-related biomarkers without correction for regression dilution bias in up to 211,754 men in UK Biobank..

	Fifths					P-trend
**Biomarkers**	**1**	**2**	**3**	**4**	**5**	
**Renal-related**						
Cystatin C						
Cases/controls	864/40587	1020/39751	1146/40235	1199/39898	1359/39448	
Model 1, HR (95% CI)	1 (Ref)	1.01 (0.92-1.10)	0.95 (0.87-1.04)	0.88 (0.81-0.97)	0.85 (0.77-0.92)	<0.0001
Model 2, HR (95% CI)	1 (Ref)	1.02 (0.93-1.11)	0.97 (0.89-1.06)	0.92 (0.84-1.00)	0.92 (0.84-1.00)	0.0183
Creatinine						
Cases/controls	999/40184	1103/39944	1120/40646	1091/39566	1277/39487	
Model 1, HR (95% CI)	1 (Ref)	1.10 (1.01-1.20)	1.09 (1.00-1.18)	1.06 (0.97-1.16)	1.12 (1.03-1.22)	0.0270
Model 2, HR (95% CI)	1 (Ref)	1.06 (0.98-1.16)	1.04 (0.95-1.13)	1.01 (0.93-1.10)	1.06 (0.98-1.16)	0.2236
Total protein						
Cases/controls	1313/36613	1098/37001	1010/36860	916/36919	849/37035	
Model 1, HR (95% CI)	1 (Ref)	0.91 (0.84-0.98)	0.88 (0.81-0.96)	0.84 (0.77-0.91)	0.82 (0.75-0.89)	<0.0001
Model 2, HR (95% CI)	1 (Ref)	0.90 (0.83-0.98)	0.88 (0.81-0.96)	0.84 (0.77-0.91)	0.81 (0.74-0.88)	**<0.0001**
Urea						
Cases/controls	869/40438	1023/40071	1055/39888	1209/39786	1435/39608	
Model 1, HR (95% CI)	1 (Ref)	1.04 (0.95-1.14)	0.99 (0.91-1.09)	1.05 (0.97-1.15)	1.12 (1.03-1.22)	0.0142
Model 2, HR (95% CI)	1 (Ref)	1.03 (0.94-1.12)	0.98 (0.90-1.07)	1.04 (0.96-1.14)	1.13 (1.04-1.24)	**0.0036**
Phosphate						
Cases/controls	1010/36954	1051/37177	1112/36824	1034/36575	978/36768	
Model 1, HR (95% CI)	1 (Ref)	1.01 (0.93-1.10)	1.11 (1.02-1.21)	1.08 (0.99-1.18)	1.11 (1.02-1.21)	0.0018
Model 2, HR (95% CI)	1 (Ref)	1.01 (0.93-1.10)	1.11 (1.02-1.21)	1.08 (0.99-1.18)	1.13 (1.03-1.24)	**0.0006**
Urate						
Cases/controls	1145/39954	1126/39940	1138/39948	1106/39930	1074/39936	
Model 1, HR (95% CI)	1 (Ref)	0.98 (0.90-1.07)	0.98 (0.91-1.07)	0.95 (0.87-1.03)	0.90 (0.83-0.98)	0.0055
Model 2, HR (95% CI)	1 (Ref)	0.97 (0.89-1.05)	0.97 (0.89-1.05)	0.94 (0.87-1.03)	0.92 (0.84-1.01)	0.0230
Microalbumin (Urine)						
Cases/controls	464/15352	404/13946	458/14160	476/14501	414/14449	
Model 1, HR (95% CI)	1 (Ref)	0.93 (0.82-1.07)	1.02 (0.89-1.16)	0.98 (0.87-1.12)	0.83 (0.73-0.95)	0.0024
Model 2, HR (95% CI)	1 (Ref)	0.94 (0.82-1.07)	1.04 (0.92-1.19)	1.03 (0.91-1.17)	0.91 (0.80-1.04)	0.1454
Potassium/creatinine ratio						
Cases/controls	960/41300	1120/41139	1158/41101	1236/41023	1279/40980	
Model 1, HR (95% CI)	1 (Ref)	0.99 (0.91-1.08)	0.96 (0.88-1.05)	0.98 (0.90-1.07)	0.99 (0.91-1.08)	0.7321
Model 2, HR (95% CI)	1 (Ref)	0.99 (0.91-1.08)	0.96 (0.88-1.05)	0.97 (0.89-1.06)	0.98 (0.90-1.07)	0.4988
Sodium/creatinine ratio						
Cases/controls	1186/41122	1252/41056	1114/41193	1113/41195	1090/41217	
Model 1, HR (95% CI)	1 (Ref)	1.09 (1.01-1.18)	0.98 (0.91-1.07)	0.98 (0.90-1.06)	0.95 (0.87-1.03)	0.0276
Model 2, HR (95% CI)	1 (Ref)	1.08 (1.00-1.17)	0.98 (0.90-1.06)	0.98 (0.90-1.07)	0.98 (0.90-1.06)	0.0846

Cox regression analysis.

Model 1: HR are stratified by region (ten UK cancer registry regions) and age at recruitment (< 45, 45 – 49, 50 – 54, 55 – 59, 60 – 64, ≥ 65 years) and adjusted for age (underlying time variable).

Model 2: HR are stratified by region and age at recruitment and adjusted for age (underlying time variable), Townsend deprivation score, ethnicity, lives with a wife or partner, BMI, smoking, physical activity, alcohol consumption, and diabetes. Full details for each covariate are provided in the statistical section.

P-values for trend from 1-SD higher concentrations analyses. Multivariable-adjusted P-values marked in boldface were statistically significant after allowing for multiple testing.

Abbreviations: BMI, body mass index; HbA1c, hemoglobin A1c; HR, hazard ratio.

Supplementary table 5. Multivariable-adjusted hazard ratios (95% CI) for prostate cancer by fifths of liver-related biomarkers without correction for regression dilution bias in up to 204,621 men in UK Biobank.

	**Fifths**					**P-trend**
**Liver-related biomarkers**	**1**	**2**	**3**	**4**	**5**	
Albumin						
Cases/controls	1287/36697	1111/37146	1043/36748	935/37016	818/37057	
Model 1, HR (95% CI)	1 (Ref)	0.96 (0.89-1.04)	1.01 (0.93-1.10)	1.00 (0.92-1.09)	1.03 (0.95-1.13)	0.3685
Model 2, HR (95% CI)	1 (Ref)	0.95 (0.87-1.03)	1.00 (0.92-1.08)	0.98 (0.90-1.07)	1.02 (0.93-1.11)	0.6438
Direct bilirubin						
Cases/controls	919/37489	1099/37485	1066/37193	1054/36825	1090/37134	
Model 1, HR (95% CI)	1 (Ref)	1.14 (1.05-1.25)	1.10 (1.00-1.20)	1.07 (0.98-1.17)	1.10 (1.01-1.20)	0.1864
Model 2, HR (95% CI)	1 (Ref)	1.13 (1.04-1.24)	1.09 (1.00-1.19)	1.06 (0.97-1.16)	1.09 (1.00-1.19)	0.2633
Total bilirubin						
Cases/controls	1046/40087	1087/39834	1136/39774	1145/39614	1159/39739	
Model 1, HR (95% CI)	1 (Ref)	1.00 (0.92-1.09)	1.03 (0.95-1.12)	1.04 (0.96-1.14)	1.08 (0.99-1.17)	0.0230
Model 2, HR (95% CI)	1 (Ref)	0.98 (0.90-1.07)	0.99 (0.91-1.08)	1.00 (0.92-1.09)	1.03 (0.94-1.12)	0.3325
Gamma glutamyltransferase						
Cases/controls	1134/39949	1151/40253	1151/39918	1107/39764	1049/39924	
Model 1, HR (95% CI)	1 (Ref)	0.95 (0.87-1.03)	0.96 (0.89-1.04)	0.95 (0.87-1.03)	0.95 (0.87-1.03)	0.2696
Model 2, HR (95% CI)	1 (Ref)	0.96 (0.88-1.04)	0.98 (0.91-1.07)	0.99 (0.91-1.08)	1.01 (0.92-1.10)	0.5565
ALT						
Cases/controls	1293/39828	1220/39873	1175/39887	1083/39948	821/40228	
Model 1, HR (95% CI)	1 (Ref)	0.96 (0.89-1.04)	0.97 (0.90-1.05)	0.97 (0.89-1.05)	0.86 (0.79-0.94)	0.0008
Model 2, HR (95% CI)	1 (Ref)	0.96 (0.89-1.04)	0.99 (0.91-1.07)	1.00 (0.92-1.09)	0.92 (0.84-1.00)	0.0682
AST						
Cases/controls	1224/40819	1153/39801	1189/39067	1087/39878	924/39542	
Model 1, HR (95% CI)	1 (Ref)	0.96 (0.88-1.04)	1.01 (0.93-1.09)	0.92 (0.85-1.00)	0.84 (0.77-0.91)	<0.0001
Model 2, HR (95% CI)	1 (Ref)	0.94 (0.87-1.02)	0.98 (0.90-1.06)	0.90 (0.83-0.98)	0.84 (0.77-0.92)	**<0.0001**

Cox regression analysis.

Model 1: HR are stratified by region (ten UK cancer registry regions) and age at recruitment (< 45, 45 – 49, 50 – 54, 55 – 59, 60 – 64, ≥ 65 years) and adjusted for age (underlying time variable).

Model 2: HR are stratified by region and age at recruitment and adjusted for age (underlying time variable), Townsend deprivation score, ethnicity, lives with a wife or partner, BMI, smoking, physical activity, alcohol consumption, and diabetes. Full details for each covariate are provided in the statistical section.

P-values for trend from 1-SD higher concentrations analyses. Multivariable-adjusted P-values marked in boldface were statistically significant after allowing for multiple testing.

Abbreviations: ALT, alanine aminotransferase; AST, aspartate aminotransferase; CI, confidence intervals; BMI, body mass index; HR, hazard ratio.

Supplementary Table 6. Multivariable-adjusted hazard ratios (95% CI) for prostate cancer restricted to blood sample from aliquot 1 after correction for regression dilution bias in up to 211,754 men in UK Biobank..

	Aliquot 1		
**Biomarkers**	**Cases/non-cases**	**HR (95% CI)**	**P-trend**
**Cardiovascular-related**			
Cholesterol	5081/180800	0.99 (0.95 - 1.04)	0.768
LDL-cholesterol	5068/180476	1.00 (0.96 - 1.05)	0.920
HDL-Cholesterol	5016/178047	1.00 (0.97 - 1.04)	0.984
Triglycerides	5074/180636	0.96 (0.91 - 1.01)	0.114
ApoA1	5005/177799	0.98 (0.95 - 1.02)	0.354
ApoB	5062/179594	1.00 (0.96 - 1.04)	0.975
C-reactive protein	5069/180381	0.97 (0.91 - 1.03)	0.279
Lipoprotein (a)	4028/144034	1.02 (0.99 - 1.05)	0.172
**Bone and joint-related**			
Vitamin D	4971/174944	1.10 (1.04 - 1.17)	**0.002**
Alkaline phosphatase	5078/180822	1.00 (0.96 - 1.03)	0.775
Calcium	5016/178129	1.03 (0.96 - 1.10)	0.421
**Diabetes-related**			
HbA1c*	-	-	-
Glucose	5009/177938	0.92 (0.85 - 1.00)	0.039
**Renal-related**			
Cystatin C	5077/180792	0.96 (0.93 - 1.00)	0.027
Creatinine	5077/180738	1.02 (0.99 - 1.06)	0.192
Total protein	5010/177935	0.89 (0.84 - 0.93)	**<0.0001**
Urea	5078/180707	1.07 (1.03 - 1.13)	**0.003**
Phosphate	5009/177807	1.14 (1.06 - 1.22)	**0.001**
Urate	5077/180661	0.97 (0.93 - 1.00)	0.081
**Liver-related**			
Albumin	5018/178164	1.01 (0.96 - 1.07)	0.613
Direct bilirubin	4754/168461	1.03 (0.99 - 1.07)	0.200
Total bilirubin	5064/180034	1.02 (0.98 - 1.06)	0.275
Gamma glutamyltransferase	5078/180710	1.01 (0.98 - 1.05)	0.460
ALT	5079/180660	0.95 (0.89 - 1.01)	0.082
AST	5065/180057	0.90 (0.85 - 0.95)	**<0.0001**

Cox regression analysis. HR are stratified by region and age at recruitment and adjusted for age (underlying time variable), Townsend deprivation score, ethnicity, lives with a wife or partner, BMI, smoking, physical activity, alcohol consumption, and diabetes. For vitamin D analyses, the model was further adjusted for month of recruitment. Full details for each covariate are provided in the statistical section.

P-values for trend from 1-SD higher concentrations analyses. Multivariable-adjusted P-values marked in boldface were statistically significant after allowing for multiple testing.

* Only 1 aliquot used for this biomarker.

Abbreviations: ALT, alanine aminotransferase; AST, aspartate aminotransferase; ApoA1, apolipoprotein A1; ApoB, apolipoprotein B; BMI, body mass index; CI, confidence intervals; HbA1c, hemoglobin A1c; HDL, high-density lipoprotein; HR, hazard ratio; LDL-cholesterol, low-density lipoperotein cholesterol; SD, standard deviation

Supplementary Table 7. Multivariable-adjusted hazard ratios (95% CI) for prostate cancer restricted to blood sample from men ≥50 after correction for regression dilution bias in up to 161,120 men in UK Biobank..

	**Incident prostate cancer**			**Prostate cancer death**		
**Biomarkers**	**Cases/non-cases**	**HR (95% CI)**	**P-trend**	**Cases/non-cases**	**HR (95% CI)**	**P-trend**
**Cardiovascular-related**						
Cholesterol	5448/150973	1.00 (0.95 - 1.04)	0.898	318/183076	1.04 (0.87 - 1.24)	0.698
LDL-cholesterol	5434/150662	1.01 (0.97 - 1.05)	0.677	318/182687	1.03 (0.86 - 1.23)	0.759
HDL-Cholesterol	5059/139358	1.00 (0.96 - 1.03)	0.830	297/169079	1.07 (0.93 - 1.23)	0.352
Triglycerides	5440/150824	0.95 (0.90 - 1.01)	0.078	318/182886	0.98 (0.79 - 1.23)	0.868
ApoA1	5048/139116	0.98 (0.94 - 1.02)	0.283	296/168806	1.07 (0.91 - 1.24)	0.426
ApoB	5426/150067	1.00 (0.96 - 1.05)	0.888	317/181879	1.03 (0.86 - 1.24)	0.759
C-reactive protein	5434/150583	0.96 (0.91 - 1.02)	0.209	318/182598	1.11 (0.89 - 1.39)	0.351
Lipoprotein (a)	4322/120063	1.02 (0.99 - 1.05)	0.187	240/145795	1.03 (0.91 - 1.16)	0.677
**Bone and joint-related**						
Vitamin D	5332/146521	1.12 (1.06 - 1.18)	**<0.001**	311/177443	0.82 (0.65 - 1.03)	0.085
Alkaline phosphatase	5444/150969	0.99 (0.96 - 1.02)	0.433	317/183069	1.11 (0.97 - 1.27)	0.120
Calcium	5059/139386	1.02 (0.95 - 1.09)	0.579	297/169120	1.00 (0.76 - 1.30)	0.972
**Diabetes-related**						
HbA1c*	5408/150145	0.97 (0.93 - 1.01)	0.171	321/181962	1.19 (1.02 - 1.39)	0.024
Glucose	5051/139253	0.91 (0.85 - 0.98)	**0.016**	296/168958	1.26 (0.97 - 1.65)	0.085
**Renal-related**						
Cystatin C	5441/150966	0.96 (0.93 - 0.99)	**0.011**	318/183055	1.02 (0.90 - 1.15)	0.794
Creatinine	5443/150885	1.02 (0.98 - 1.05)	0.304	318/182974	0.94 (0.82 - 1.07)	0.345
Total protein	5053/139239	0.88 (0.84 - 0.93)	**<0.001**	296/168955	1.14 (0.92 - 1.41)	0.221
Urea	5444/150861	1.07 (1.02 - 1.12)	**0.004**	318/182949	0.94 (0.79 - 1.13)	0.518
Phosphate	5052/139161	1.13 (1.05 - 1.21)	**0.001**	296/168851	1.17 (0.87 - 1.59)	0.296
Urate	5442/150800	0.96 (0.92 - 0.99)	**0.016**	317/182864	0.95 (0.82 - 1.10)	0.503
Creatinine (Urine)	5610/155510	1.08 (0.98 - 1.20)	0.127			
Microalbumin (Urine)	2216/72408	0.96 (0.90 - 1.02)	0.167			
Potassium/creatinine ratio	5601/155200	0.95 (0.85 - 1.06)	0.369			
Sodium/creatinine ratio	5602/155347	0.89 (0.79 - 1.01)	0.081			
**Liver-related**						
Albumin	5061/139432	1.02 (0.96 - 1.08)	0.545	297/169174	1.01 (0.80 - 1.29)	0.908
Direct bilirubin	5095/141078	1.03 (0.99 - 1.07)	0.151	296/170589	0.85 (0.71 - 1.02)	0.086
Total bilirubin	5426/150331	1.03 (0.99 - 1.07)	0.182	318/182280	0.83 (0.71 - 0.98)	0.028
Gamma glutamyltransferase	5445/150887	1.01 (0.97 - 1.04)	0.761	318/182963	1.14 (0.99 - 1.32)	0.077
ALT	5445/150869	0.94 (0.89 - 1.00)	0.052	318/182931	1.04 (0.82 - 1.32)	0.753
AST	5430/150396	0.90 (0.85 - 0.94)	**<0.001**	318/182339	1.01 (0.83 - 1.23)	0.950

Cox regression analysis. HR are stratified by region and age at recruitment and adjusted for age (underlying time variable), Townsend deprivation score, ethnicity, lives with a wife or partner, BMI, smoking, physical activity, alcohol consumption, and diabetes. For vitamin D analyses, the model was further adjusted for month of recruitment. Full details for each covariate are provided in the statistical section.

P-values for trend from 1-SD higher concentrations analyses. Multivariable-adjusted P-values marked in boldface were statistically significant after allowing for multiple testing.

Abbreviations: ALT, alanine aminotransferase; AST, aspartate aminotransferase; ApoA1, apolipoprotein A1; ApoB, apolipoprotein B; BMI, body mass index; CI, confidence intervals; HbA1c, hemoglobin A1c; HDL, high-density lipoprotein; HR, hazard ratio; LDL-cholesterol, low-density lipoperotein cholesterol; SD, standard deviation

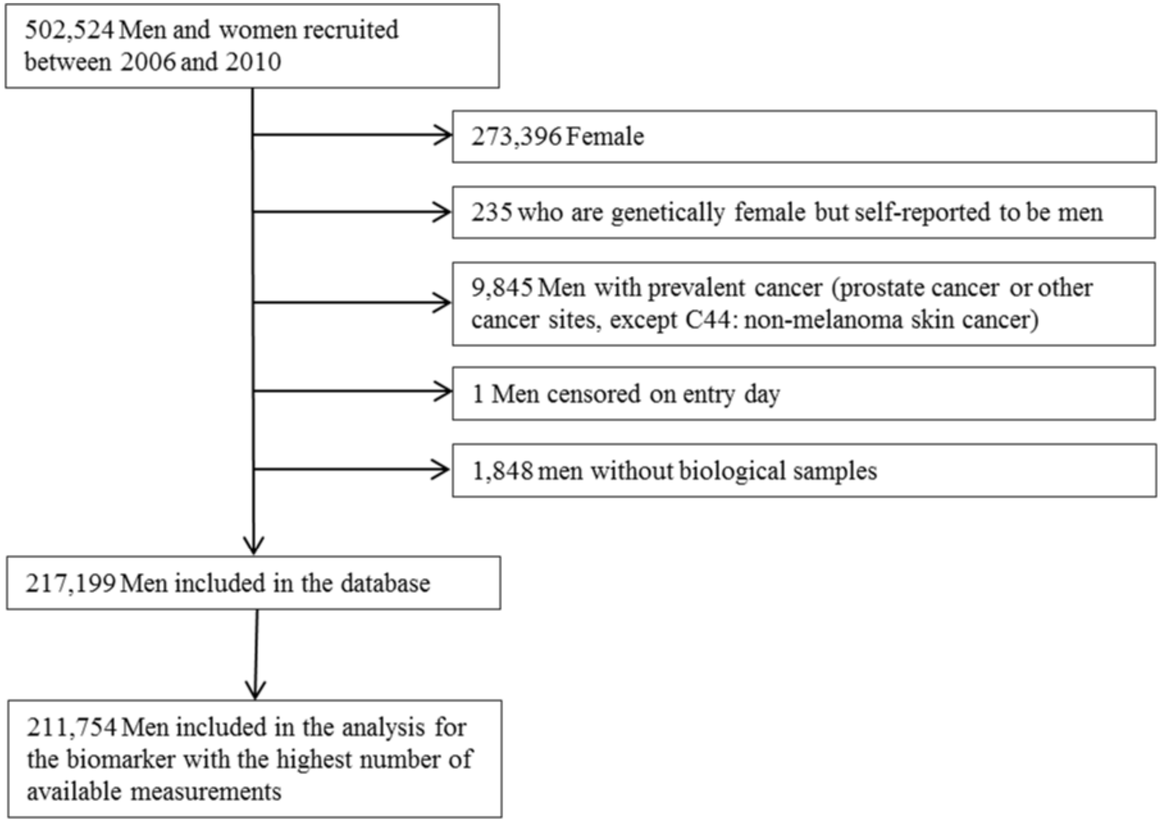

Supplementary Figure 1. Selection criteria of the study participants in UK Biobank. The final number of participants vary in each biomaker.
